# Application of Enoki Mushroom (*Flammulina Velutipes*) Stem Wastes as Functional Ingredients in Goat Meat Nuggets

**DOI:** 10.3390/foods9040432

**Published:** 2020-04-04

**Authors:** Dipak Kumar Banerjee, Arun K. Das, Rituparna Banerjee, Mirian Pateiro, Pramod Kumar Nanda, Yogesh P. Gadekar, Subhasish Biswas, David Julian McClements, Jose M. Lorenzo

**Affiliations:** 1 Eastern Regional Station, ICAR-Indian Veterinary Research Institute, Kolkata 700 037, India; dipakkumarbanerjee23@gmail.com (D.K.B.); npk700@gmail.com (P.K.N.); 2 Department of Livestock Products Technology, West Bengal University of Animal and Fishery Sciences, Kolkata 700 037, India; rituparnabnrj@gmail.com (R.B.); lptsubhasish@gmail.com (S.B.); 3 Centro Tecnológico de la Carne de Galicia, Adva. Galicia n° 4, Parque Tecnológico de Galicia, San Cibrao das Viñas, 32900 Ourense, Spain; mirianpateiro@ceteca.net; 4 ICAR-Central Sheep and Wool Research Institute, Avikanagar, Jaipur 304 501, India; yogirajlpt@gmail.com; 5 Department of Food Science, University of Massachusetts, Armhrest, MA 01003, USA; mcclemen@umass.edu

**Keywords:** enoki mushroom, physicochemical properties, antioxidant activity, dietary fiber, goat meat nuggets quality, sensory characteristics

## Abstract

The impact of different amounts (2%, 4% and 6%) of enoki (*Flammulina velutipes*) mushroom stem waste (MSW) powder on the physicochemical quality, color and textural, oxidative stability, sensory attributes and shelf-life of goat meat nuggets was evaluated. These mushroom by-products (MSW powder) contained a good source of protein (13.5%), ash (8.2%), total phenolics content (6.3 mg GAE/g), and dietary fiber (32.3%) and also exhibited the potential to be strong antioxidants, due to their good metal chelating ability (41.3%), reducing power (60.1%), and free radical scavenging activity (84.2%). Mushroom stem waste improved (*p* < 0.05) the emulsion stability, dietary fiber, ash and phenolics content of nuggets compared to control. Although no significant differences (*p* > 0.05) in expressible water and textural properties were observed among the formulations, but MSW powder improved the water holding capacity and slightly decreased the hardness. Further, the inclusion of MSW significantly (*p* < 0.05) improved the oxidative stability and shelf-life of treated nuggets by reducing lipid oxidation during the nine-day storage period. Again, the inclusion of MSW did not negatively affect the color and sensory attributes of treated meat nuggets. Overall, our results suggest that enoki mushroom stem waste (4%) can be used as a value-added functional ingredient to produce nutritionally improved and healthier meat products.

## 1. Introduction

Mushrooms, due to their good nutritional attributes and richness in high-quality proteins, dietary fibers, vitamins, minerals, and phenolic compounds, are considered to be a healthy food product [1,2]. Enoki mushrooms (*Flammulina velutipes*), popularly known in different countries as “golden needle”, “winter”, “lily” or “velvet stem” mushrooms, are widely recognized for their good nutritional value and desirable taste attributes [3,4]. Several compounds including carbohydrates, protein, lipids, glycoproteins, phenols, and sesquiterpenes have been isolated from different parts of this mushroom [5]. Enoki mushrooms have also been known to exhibit good antioxidant, anti-inflammatory, immunomodulatory, anti-cancer and cholesterol-lowering activities [2,6]. The cultivated variety of this mushroom has a pure white bean sprout, a velvety stem, and a tiny snowy-white cap, whereas the wild variety may be orange to brown with a larger, shiny cap [7]. The stem base and other parts of the mushroom are removed during harvesting and these leftovers either go to landfills or are used as compost [8].

There is a growing interest in the application of plant-based waste materials as functional food ingredients in meat products, as they are a rich source of dietary fiber and several other bioactive compounds like vitamins, minerals, and polyphenols [9,10,11,12]. These dietary fibers, in combination with phenolic compounds, form antioxidant dietary fibers (ADFs) [13,14] which can be used as dietary supplements to improve gastrointestinal health, or as technical ingredients to inhibit lipid oxidation in foods, thereby extending their shelf-life [15,16]. As far as enoki mushrooms are concerned, its extract is reported to have strong antioxidant potential, with a high 2, 2-dipheny-1-picrylhydrazyl (DPPH) radical scavenging activity and metal chelating ability. Being a rich source of dietary fiber, the extract may reduce triacylglycerol, total cholesterol, and low-density lipoprotein levels in the blood due to a variety of mechanisms [4].

In summary, mushroom powder extracts have numerous nutritional attributes such as low calorie-density, healthy lipid profile, high fiber, protein and phenolic contents that make them suitable for incorporation as functional food ingredients in a variety of food products [17]. Previously, powdered mushroom extracts have been used to fortify a variety of foods, including biscuits, cookies, crackers, and cakes [18]. There have also been a limited number of studies on the incorporation of various kinds of mushroom extract into meat products. For instance, oyster mushrooms (*Pleurotus ostreatus*) have been investigated as a substitute for pork meat in Thai glutinous fermented sausage [19]. Reports on the impact of dried portobello mushroom (*Agaricus bisporus*) on the quality characteristics of a dry spicy sausage, sucuk [20], texture and structure of meat emulsions [1] and physicochemical and sensory properties of cold-stored beef patties [21], effects of oyster mushroom (*Pleurotus sajor-caju*) on the color, texture, cooking characteristics, and fiber content of chicken patties [22] are also available. Besides, enoki MSW has been used as a potential substitute for antibiotics in organic egg production by chickens [8] and to enhance the growth and health status of broiler chickens [3]. To the authors’ knowledge, no study has previously been carried out on the utilization of enoki MSW as a functional food additive in processed meat products. The current study was designed to analyze the dietary fiber content and antioxidant properties of enoki MSW powder, and then to evaluate its potential as a functional ingredient in goat meat nuggets.

## 2. Materials and Methods

### 2.1. Materials and Reagents

Mushroom stem waste was collected from the harvesting and processing area of an enoki mushroom facility, cleaned properly for extraneous dirt, if any, and then dried in an oven (Static Oven, Instrumentation India, Kolkata) at 50 °C for 8 h. The dried MSW sample was then ground using a grinder (Kenstar, Mumbai, India) into powder (0.01 mm), which was then used as a functional ingredient in the goat meat product formulation. The proximate composition and antioxidant activity of this powder was also quantified. Goat meat (leg part) was procured from a supermarket and then kept in a freezer (−18 °C) until further processing. Chemical reagents such as methanol, sodium carbonate, 2-thiobarbituric acid (TBA), trichloroacetic acid (TCA), α-amylase, protease, amyloglucosidase, Folin-Ciocalteu (F-C) reagents, and gallic acid were procured from Sigma-Aldrich (Mumbai, India). Other chemicals and reagents were of analytical grade (SRL, Mumbai, India). 

### 2.2. Chemical Composition and Extract Preparation of Mushroom Stem Waste

For chemical composition such as moisture, protein, fat, and ash content, duplicate MSW samples were analyzed based on the methods of the Association of Official Analytical Chemists [23]. The enzymatic-gravimetric process was used for dietary fiber estimation [24]. Briefly, different enzymes such as heat-stable α-amylase, protease and amyloglucosidase were used for sequential enzymatic digestion of MSW samples after its proper dispersion in phosphate buffer solution. Then, after filtration of the insoluble dietary fiber (IDF), warm distilled water was used to wash the residue. Ethanol (95%) was used for the precipitation of soluble dietary fiber (SDF) from the combined solution of filtrate and washings. The weight was noted after proper drying of residue in an oven (Static Oven, Instrumentation India, Kolkata) at 50 °C. These IDF and SDF residues later were also analyzed for protein and ash content. Both IDF and SDF fractions were combined for total dietary fiber (TDF) calculation.

For the preparation of MSW extract, water was used as a solvent [9]. Briefly, 20 g of MSW powder was accurately weighed into a conical flask. To this, 1000 mL of solvent was added and the whole content was held at room temperature (29 ± 1 °C) for 10 h, stirring frequently with a glass rod. The mixture was shaken at a constant rate (500 rpm) using a shaker, vortexed at high speed for 10 min, and finally centrifuged (REMI NEYA 8, Kolkata, India) at 5000× rpm for 10 min. The content of extract was then passed through a Whatman filter paper No. 1 (HiMedia^®^, Mumbai, India). The resulting extract was kept in a container and stored at 2 °C for further studies. The aqueous extracts obtained from repeated extractions were analyzed for total phenolic content (TPC), DPPH radical scavenging activity, ferrous ion chelating ability and reducing power assays. The efficacy of the extract was determined based on the dry weight of the mushroom powder.

### 2.3. Antioxidant Activity of Mushroom Stem Waste

#### 2.3.1. Total Phenolics Content

The total phenolics content (TPC) of MSW was estimated using the Folin-Ciocalteu method. Briefly, 0.1 mL aqueous extract was properly mixed with 0.75 µL of F-C reagent and then a final volume of the above mix was increased ten-times using deionized water. Then, a sodium carbonate solution (750 µL) was added in each test tubes after 5 min and these tubs were incubated (in the dark) for 90 min at room temperature. The absorbance of test samples at 725 nm was taken using a spectrophotometer (Thermo Scientific, Wilmington, NC, USA) against a blank. Different concentrations of gallic acid were used for preparation of a standard curve and the TPC was calculated as gallic acid equivalents (GAE) in mg/g dry weight basis of MSW.

#### 2.3.2. DPPH Radical Scavenging Activity

The method developed by Shimada et al. [25] was used for the measurement of DPPH radical scavenging activity. Briefly, 4 mL of methanol was added to a 1 mL extract from the MSW powder in a test tube and then 1 mL of 0.2 µM DPPH methanol solution was added and mixed. Samples were then incubated for 30 min and later the absorbance at 517 nm was measured using a spectrophotometer (Thermo Scientific, Wilmington, NC, USA). The scavenging activity was calculated by the following formula:

(1) $$\mathrm{Scavenging}\;\mathrm{activity}\left(\%\right)=\left[1-\left(\frac{absorbace\;of\;extract}{absorbance\;of\;control}\right)\times 100\%\right]$$

#### 2.3.3. Ferrous Ion Chelating Ability

The ferrous ion chelating ability of the MSW extract was measured using the procedure outlined by Dinis et al. [26]. Briefly, 3.7 mL methanol and 0.1 mL of 2 *µ*M FeCl_2_, MSW extract (0.1 mL) were mixed properly and then held for 30 s before adding 0.1 mL of 5 mM ferrozine solution. The mixture was then kept for 10 min at room temperature for incubation purposes. Finally, the absorbance using a spectrophotometer (Thermo Scientific, Wilmington, NC, USA) was recorded at 562 nm. The formula for calculating the ferrous ion chelating ability is as follows:

(2) $$\mathrm{Chealting}\;\mathrm{ability}\left(\%\right)=\left[1-\left(\frac{\mathrm{absorbace}\;\mathrm{of}\;\mathrm{extract}}{\mathrm{absorbance}\;\mathrm{of}\;\mathrm{control}}\right)\times 100\%\right]$$

#### 2.3.4. Ferric Reducing Antioxidant Power

The ferric reducing antioxidant power (FRAP) of the MSW extract was determined based on the method described by Madane et al. [9]. Briefly, 2.5 mL of extract taken in a 10 mL test tube was added with 2.5 mL of phosphate buffer (0.2 M, pH 6.6) and 2.5 mL of 1% (*w*/*v*) potassium ferricyanide. Then, 2.5 mL of 10% TCA was added after the mixture was kept for incubation at 50 °C for 20 min. After that, 2.5 mL of deionized water and 0.5 mL of ferric chloride (0.1% *w*/*v*) were mixed with 2.5 mL of the supernatant. Then, absorbance of samples was taken at 700 nm using a spectrophotometer (Thermo Scientific, Wilmington, NC, USA) and expressed as a percentage.

### 2.4. Preparation of Goat Meat Nuggets

Four formulations (control and treatments-T2, T4 and T6) of goat meat nuggets were prepared following the standard method described by Das et al. [27]. The first batch was considered as a control (meat without MSW powder), whereas in the case of T2, T4 and T6 formulations, MSW powder at 2.0%, 4.0% and 6.0% was included, respectively, replacing an equal percent of goat meat. Therefore, the total weight was 100 g with salt, condiments, spice mix, oil and wheat flour (Table 1).

Before processing, the frozen goat meat was thawed, cut into small cubes, and then minced using a meat mincer (Stadler, Mumbai, India). Meat emulsion was prepared separately for each group (control, T2, T4 and T6) by thoroughly mixing goat meat cubes with other ingredients (salt, sugar, phosphate, and nitrite) in a bowl chopper. During chopping, ice flakes were added to prevent excessive heating. Condiments, dry spice mix, and fine wheat flour were then added and chopped continuously until all the ingredients were uniformly mixed. About 500 g of emulsion from each formulation was placed in a mold, and cooked for 40 min with a steam-cooker (Stadler, Mumbai, India). The cooked meat blocks obtained were then sliced and cut into small pieces. Nugget samples of different formulations were then analyzed for various parameters (physicochemical, textural, colour) and also aerobically packed into low density polyethylene pouches and stored for up to nine days at 4 ± 1 °C for lipid oxidation study.

### 2.5. Analysis of Meat Products

#### 2.5.1. pH, Emulsion Stability and Cooking Yield

The pH of the meat emulsion and nugget samples were measured after blending a 10 g sample with 50 mL of deionized water for a minute in a homogenizer (Omni International, Kennesaw GA, USA) and then using a digital pH meter. For emulsion stability, 25 g of emulsion was placed in a polypropylene bag and heated in a thermostatically controlled water bath for 20 min at 80 °C. The cooked mass was then cooled and weighed after draining out the exudate. For cooking yield, the weight of each meat block before and after cooking was recorded. The cooking yield was calculated and expressed as a percentage.

(3) $$\mathrm{Cooking}\;\mathrm{yield}\;\left(\%\right)=\frac{\mathrm{Weight}\;\mathrm{of}\;\mathrm{cooked}\;\mathrm{meat}\;\mathrm{block}}{\mathrm{Weight}\;\mathrm{of}\;\mathrm{raw}\;\mathrm{meat}\;\mathrm{block}}\times 100$$

#### 2.5.2. Expressible Water

The percentage of expressible water which indicates the water holding capacity of cooked processed meat, was measured using the method of Madane et al. [9]. About 5 g of nugget sample was taken on two layers of Whatman No. 1 filter paper and placed the filter paper with sample in a 50 mL centrifuge tube for centrifugation (REMI NEYA 8, Kolkata, India) at 1500 rpm for 5 min. The sample was re-weighed after centrifugation and expressible water was calculated according to the following equation:

(4) $$\mathrm{Expressible}\;\mathrm{water}\;\left(\%\right)=\frac{\left(\mathrm{initial}\;\mathrm{weight}-\mathrm{final}\;\mathrm{weight}\right)}{\mathrm{initial}\;\mathrm{weight}}\times 100$$

#### 2.5.3. Total Phenolics Content in Meat Nuggets

Total phenolics content of meat nuggets was analyzed calorimetrically using F-C reagent. Briefly, the meat nugget sample (5 g) was homogenized at 3000 rpm for 2 min in a tube with 15 mL of distilled water. The mixture was shaken vigorously two to three times after adding 9 mL chloroform to separate the lipids. The F-C reagent (500 µL) was added to 1 mL aliquot of the diluted sample (1:4, *v*/*v*) followed by the addition of 1 mL of the sodium carbonate solution (5%). After proper mixing, samples were incubated for 1 h at room temperature and then vortexed. Then absorbance was taken at 700 nm. The results were expressed as mg gallic acid equivalents (GAE)/g of the dry weight of the meat nuggets.

#### 2.5.4. Texture Profile Analysis

The textural properties such as hardness, springiness, gumminess, cohesiveness and chewiness of the control and MSW powder treated goat meat nuggets were measured with the help of a texture analyzer (Stable Micro System Model TA.XT 2i/25, Surrey, UK). The measurement was carried out using central cores of five pieces of each meat sample (2 cm × 2 cm × 2 cm). Each sample was compressed twice (80% of the original height and 2 mm/s crosshead speed) with the help of a compression probe (P 75).

#### 2.5.5. Instrumental Color Attributes

Various color attributes of the meat nuggets were measured using a Hunter Color Lab instrument in which the Hunter L*, a*, and b* values were determined. L* denoted pure white (100)/pure black (0), a* +redness/-greenness and b* +yellowness/-blueness. A light trap/black glass and white tile was used for calibration of instrument. The color attributes of the nugget surfaces were analyzed at three different points.

#### 2.5.6. Sensory Evaluation

The sensory attributes, including appearance, flavor, texture, juiciness, and overall acceptability, of meat nuggets were evaluated using an eight-point descriptive scale, where 8 = excellent and 1 = extremely poor [28]. The purpose of the experiments was clearly explained to the panelists without disclosing the much about samples’ identity. The panelists evaluated the sample based on a sensory preformed provided after coding with number and warming in a microwave oven for 1 min. Water was provided to the panelists to rinse their mouths during evaluation.

### 2.6. Lipid Oxidation in Meat Products

#### 2.6.1. Peroxide Values

The peroxide values (PV) of the nugget samples were determined following the procedure described by Koniecko [29] with slight modifications. Briefly, for filtrate preparation, anhydrous sodium sulfate and chloroform was used to blend the meat sample (5 g). 2 mL of saturated potassium iodide solution were added to a mixture of 25 mL filtrate and 30 mL glacial acetic acid. After proper mixing for about 2 min, 100 mL of distilled water and 2 mL of fresh 1% starch solution were added and the mixture was titrated immediately with 0.1 N sodium thiosulphate until the end point was reached (the non-aqueous layer turned colorless). The PV of the meat sample was calculated and expressed in meqO_2_/kg of the sample.

#### 2.6.2. Thiobarbituric Acid Reacting Substances

The measurement of thiobarbituric acid reacting substances (TBARS) was carried out to measure the lipid oxidation in the meat nugget during storage [30]. Briefly, the TCA extract was prepared after triturating samples with 20% pre-cooled TCA (25 mL). To 3 mL TCA extract (filtrate), 3 mL of TBA reagent (5 mM) was added and then cooled in running tap water after boiling in a thermostatically controlled water bath at 70 °C for 35 min. Similarly, a blank was prepared by adding and properly mixing 3 mL of 10% TCA and 3 mL of the 5mM TBA reagent. Then, absorbance was recorded at a 532 nm. The TBARS value was expressed as mg malonaldehyde (MDA) per kg of meat sample.

### 2.7. Statistical Analysis

Measurements of all the parameters were performed in duplicate and this study was conducted thrice. The data collected from this study were analyzed by a SPSS software (version 20.0, Armonk, NY, USA). The normal distribution and variance homogeneity were previously assessed (Shapiro-Wilk). In the case of the lipid oxidation study, the data were analyzed using two-way ANOVA with treatments (control, T2, T4 and T6) and storage time (0, 3, 6, 9 days) as the main effects (4 × 4 factorial design). To find out the impact of MSW on various parameters, Duncan’s multiple range tests were applied. The values are presented here as the mean and standard error, and significance differences were identified at the 95% confidence level (P < 0.05).

## 3. Results and Discussion

### 3.1. Proximate Composition and Dietary Fiber Content of Mushroom Stem Waste

The proximate composition and dietary fiber content of enoki MSW powder is presented in Table 2. The MSW powder had high moisture (12.9%), protein (13.5%) and ash (8.2%), but a relatively lower fat content (1.5%). Available reports indicate that dried mushrooms contain relatively high moisture levels, depending on the species and other factors [31], more than 25% protein, less than 3% crude fat, and around 50% total carbohydrate [32]. Further, the ash content in mushrooms typically ranges between 5%–12% of dry matter. The composition of enoki MSW analyzed by other researchers [3,8,33] varied between 12.75% and 18.42% for crude protein, fat from 1.5% to 2.94% and ash between 6.33% and 11.6%, which is well within the level found in our study (Table 2). In this regard, variation in proximate composition could be due to differences in harvesting methods and stages of maturity of mushroom, soil types and environmental factors [34].

In this study, the SDF and IDF contents of the enoki MSW were found to be 17.3% and 15.1%, respectively (Table 2). Many researchers have reported the SDF and IDF content of mushrooms ranging between 22.4–31.2% and 4.2–9.2% of dry weight, respectively [35]. The TDF (32.3%) content determined in our study is fairly similar to that reported for raw enoki mushrooms (29.3%) [36]. In a comparison of different kinds of mushrooms, Yang, Lin, & Mau [37] found a higher dietary fiber content in enoki mushrooms than in shiitake or oyster mushrooms. The ability of enoki mushrooms to lower cholesterol and blood pressure levels may be partly attributed to their relatively high dietary fiber content [4]. Moreover, these dietary fibers may help in the formulation of low-calorie, low-fat and high-fiber meat products, due to their ability to form gel networks that hold water and modulate texture.

### 3.2. Antioxidant Activity of Mushroom Stem Waste Extract

The total phenolics content of the enoki mushroom stem extract was 6.26 mg GAE/g dry weight, which was determined using gallic acid as a standard (Table 2). The antioxidant potential of this extract was assessed using a number of assays: the DPPH assay was used to measure the free radical scavenging ability [38]; the FRAP assay was used to measure the reducing power (Fe^3+^ to Fe^2+^); and, the iron-binding assay was used to measure the ability to chelate transition metal irons [39]. The enoki mushroom stem extract was found to have strong antioxidative potential: 84.2% DPPH scavenging; 60.1% reducing power; and 41.3% of ferrous ion chelating ability (Table 2).

In fact, phenolic compounds that are found naturally in mushrooms have antioxidant activity due to their hydrogen-donating and singlet oxygen-quenching properties [40,41]. The antioxidant properties of the enoki mushroom extract can be attributed to a number of antioxidant constituents, including p-coumaric acid, ellagic acid [42], gallic acid, pyrogallol, chlorogenic acid, caffeic acid, ferulic acid, and quercetin [40]. The phenolics content of mushrooms has been shown to be positively correlated with the results of the DPPH assay and other antioxidant assays [4]. Previous studies have shown that enoki mushrooms have higher phenolics contents, ferric reducing powers, and ferrous chelating activities than other mushrooms [43]. Taken together, these studies suggest that enoki mushroom extracts are a good source of natural antioxidants.

### 3.3. Physicochemical Properties and Proximate Composition of Fortified Goat Meat Nuggets

The pH of the meat emulsion without MSW powder (control) was the lowest among all the treatments (Table 3). The addition of the mushroom powder significantly (*p* < 0.05) increased the pH. The meat emulsion containing 6.0% MSW had the highest pH value (6.44). Our results are in agreement with the findings of Bao, Ushio, & Ohshima [44], who earlier reported that the addition of enoki mushroom extracts to beef and fish slightly increased the pH of their products, although the increase was statistically non-significant. The increase in pH of products could be due to the abundance of basic amino acids in comparison to acidic amino acids with addition of enoki mushroom powder [45], as well as the natural buffering capacity of the mushroom proteins [46].

There was a significant (*p* < 0.05) increase in the emulsion stability of the treated meat nuggets, whereas cooking loss (%) reduced significantly with an increase in level of MSW powder incorporation, compared to control. On the other hand, the expressible water (%) decreased with increased powder level, indicating an improvement in water holding capacity, although this change was not statistically significant (*p* > 0.05). The improved emulsion stability and reduced cooking loss were probably because of the higher TDF (32.3%) content of enoki MSW which enhanced the oil absorption and water retention properties of the meat emulsion [47]. The improvement in water binding and fat retention in meat products, upon the addition of dietary fiber from several sources, have been reported by various researchers [16,47].

The TPC of meat nuggets increased significantly (*p* < 0.05) with the increasing level of MSW powder, rising from 0.14 to 0.96 mg GAE/g dry weight of product as the MSW content increased up to 6.0%. This is in agreement with the findings of different researchers who reported significantly increased TPC of meat nuggets upon addition of guava powder [16] and dragon fruit peel powder [9]. The increased phenolics content in treated meat nuggets could be due to addition of powdered enoki mushroom stem extract, which is reported to possess several bioactive phenolic and polyphenolic compounds [40,42,43].

Incorporation of MSW powder did not have a significant effect on the moisture, protein, and fat contents of the meat nuggets, but it led to a significant (*p* < 0.05) increase in the ash and dietary fiber contents (Table 3). These effects can be attributed to the presence of relatively high levels of minerals and dietary fibers in the mushrooms. It has been reported that this kind of mushroom is not only rich in potassium and phosphorus [43,48], but also contains several other minerals in minor amounts such as sulfur, sodium, copper, iron, and zinc [31]. In addition, the powdered enoki mushroom extract contained higher levels of TDF (32%), which would contribute to the fiber content of the final meat product.

### 3.4. Textural and Color Attributes of Meat Nuggets

The effects of incorporating powdered enoki MSW on the textural and color attributes of the meat nuggets is presented in Table 3. Although various textural parameters like hardness, springiness, cohesiveness, and gumminess of the nuggets decreased slightly, the reduction was not statistically significant. However, the chewiness of treated nuggets decreased significantly (*p* < 0.05) with an increased level of mushroom powder addition. Our results are in tandem with the findings of Choe et al. [49] who added enoki mushroom extracts to emulsion-type sausages and obtained similar results. Other researchers have shown that replacing chicken meat with 25% or 50% oyster mushroom (*P. sajor-caju*) reduced the hardness and improved the textural parameters of chicken patties [22]. The addition of portobello mushroom powder has been shown to increase the hardness and cohesiveness of model meat emulsions up to 3%, but then decrease their textural attributes at higher addition levels [1]. The textural properties of cooked meat products containing plant materials are related to the gelation of the myofibrillar proteins from the meat [50], as well as the biopolymer networks formed by the dietary fibers from the plants [47]. The addition of dietary fiber may have influenced the gelation of the meat proteins, thereby decreasing the gel strength and leading to a softer texture [14,15,47].

The color of any fresh or processed food product plays an important role in influencing the decision of consumers [51]. It is, therefore, imperative that any functional ingredient added to improve the nutritional properties of a meat product does not cause undesirable changes in its appearance. Increasing the amount of powdered enoki mushroom extract in nugget formulations increased the lightness (L*) and reduced redness (a*), but did not change their yellowness (b*) values (Table 3). No statistical differences (*p* > 0.05) in the lightness and redness of the meat nuggets containing 2.0% and 4.0% mushroom extract were observed compared to the control group. However, there was a significant increase in lightness and reduction in redness (*p* < 0.05) in nuggets samples prepared with 6.0% MSW. This might be a result of the dilution of the meat protein due to the addition of mushroom powder as a percentage of the meat and the white color of mushroom powder. Moreover, there may have occurred an increase in the degree of light scattering by the particles in the mushroom powder, which caused the meat product to become lighter. Similar results were reported by Choe et al. [49] in enoki mushroom powder added to emulsion-type sausages. In another study, it was reported that adding portobello mushroom extracts to a meat emulsion led to a decrease in L* value and an increase in a* value [1], which suggests that the effects may be system dependent.

### 3.5. Sensory Characteristics of Meat Nuggets

The sensory parameters of goat meat nuggets containing different levels of powdered enoki mushroom extract are presented in Table 4. There was no significant difference (*p* > 0.05) in the individual sensory attributes of the meat nuggets regardless of the level of mushroom waste used. However, the appearance, flavor, and overall acceptability of nuggets decreased slightly with the addition of 6.0% MSW powder. Unlike other mushroom species, enoki mushroom is reported to have a very mild and delicate taste [52]. This is desirable and may be a beneficial attribute for many food applications, as it does not strongly alter or mask the expected sensory attributes of the original product. Few reports available in this regard suggest that enoki mushrooms contain high levels of free amino acids, which are associated with umami or monosodium glutamate-like, sweet, and bitter tastes that are often perceived favorably by the consumers [37]. The white color of the mushrooms may also be beneficial because it does not alter the overall hue of the final meat product, but may slightly decrease its lightness.

In this regard, researchers have shown that supplementation of pork patties with the ground, white jelly mushrooms at 10%, 20%, and 30% by weight did not affect the liking of appearance, color, flavor, or texture [53], but the acceptability was better at a 10% level. In another study, Myrdal Miller et al. [54] reported that the addition of ground white button mushrooms to ground meat did not have a major impact on their perceived quality attributes. Taken together, these results suggest that mushrooms can be used as a healthy substitute for meat products without adversely impacting their desirable appearance or flavor.

### 3.6. Lipid Oxidation of Meat Nuggets During Storage

**Figure 1:**
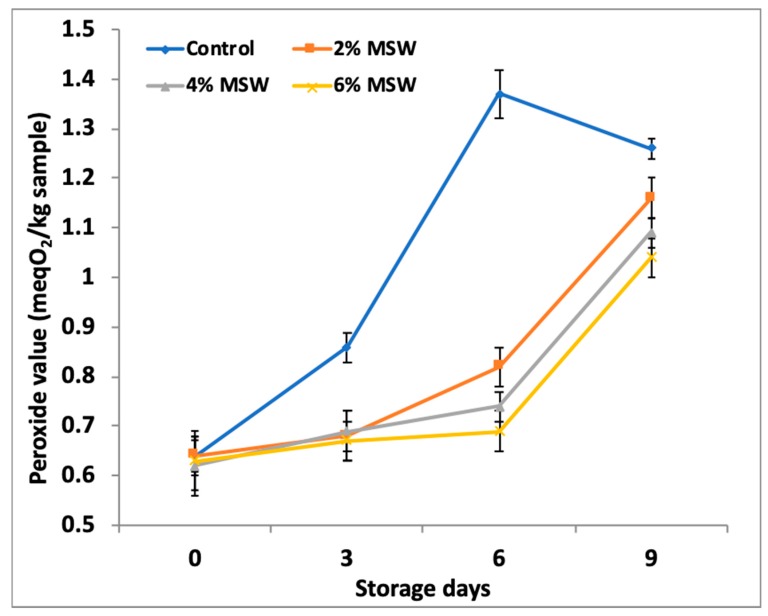
Effect of mushroom stem waste (MSW) powder on peroxide values of goat meat nuggets during storage.

**Figure 2:**
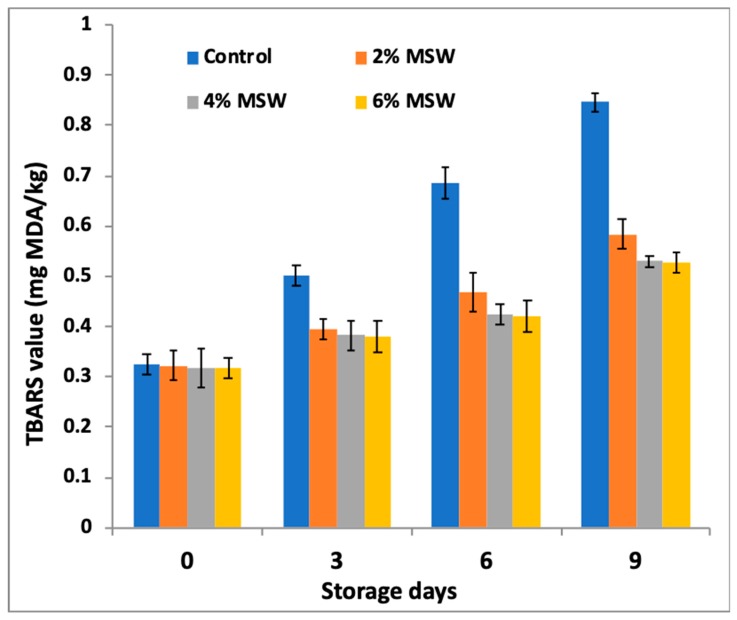
Effect of mushroom stem waste (MSW) powder on TBARS values of goat meat nuggets during storage.

The impact of the powdered MSW on the oxidative stability of the meat nuggets was determined by measuring the primary (PV) and secondary (TBARS) lipid oxidation products over time and is presented in Figure 1 and Figure 2. The treated meat nuggets (T2, T4, and T6) had significantly (*p* < 0.05) lower peroxide and TBARS values than the control group, although there was no significant difference (*p* > 0.05) between the levels of powder addition.

Hydroperoxides are primary products of lipid oxidation, hence PV are important to know the extent of initial lipid oxidation in meat samples. The results depicted in Figure 1 indicated that the initial PV (0.64 meqO_2_/kg) of control nuggets increased to 1.21 meqO_2_/kg after nine days of storage, which was significantly higher (*p* < 0.05) compared to treated nuggets. Similarly, results suggested that the control samples underwent noticeable lipid oxidation during the first six days of refrigerated storage and reached maximum PV at the end of the primary auto-oxidation. After six days of storage, the hydroperoxides formed might have gone through the decomposition to form secondary lipid oxidation products [55]. Although oxidation in the control was more intense compared to the treated samples, a decline was observed on day six. This indicates that, after the induction period, the decomposition rate of the hydroperoxides was faster than the production rate [56].

The control nugget had an initial TBARS value of 0.32 and it reached 0.85 mg MDA/kg on the ninth day of storage study, whereas TBARS value in treated nuggets with 2–6% MW increased from 0.32–0.58 mg MDA/kg (Figure 2). There was an increase in TBARS values during storage irrespective of treatment but at a slower rate in treated nuggets compared to the control, indicating the effectiveness of MSW in inhibiting lipid oxidation during the storage. MSW besides supplementing the dietary fiber to goat meat nuggets was found to retard lipid peroxidation in the product during refrigerated storage. Although the secondary reaction products showed an upward trend in all the samples as storage days progressed, a considerably slower rate was observed in the samples containing the mushroom extracts. These results suggest that the enoki mushroom extracts were effective antioxidants that were able to retard lipid peroxidation in treated goat meat nuggets during refrigerated storage study for up to nine days.

In general, mushrooms have been shown to contain a variety of different antioxidant substances that make them effective at inhibiting lipid oxidation [4,7,31,57]. In an earlier section, we showed that the enoki mushroom extracts had strong reducing power, high scavenging activity, and good iron-binding ability (Table 2). These antioxidant effects may be attributed to the relatively high phenolic, dietary fiber, ergothioneine, vitamin C, and nucleotides content [7]. Antioxidant effects have also been reported when winter mushroom extract is added to beef and fish products [44] and emulsion-type sausages [49]. Enoki mushroom extracts have also been shown to prevent discoloration and lipid oxidation in fish and melanosis in crustaceans during postmortem storage [44,58].

## 4. Conclusions

Our study has shown that enoki mushroom (*F. velutipes*) stem waste is a good source of bioactive ingredients, such as dietary fibers and phenolics. The extract of MSW powder exhibited good antioxidant potential, which was attributed to its strong free radical scavenging activity, ferric reducing power, and metal chelating ability. The incorporation of this extract into a model meat product (goat meat nuggets) increased its dietary fiber and ash content, which may have nutritional benefits. Moreover, the mushroom extract improved the cooking yield and did not adversely affect the appearance or texture of the final product. Finally, the mushroom extract significantly improved the shelf-life of the meat products, which was mainly attributed to its ability to inhibit lipid oxidation during storage. Therefore, enoki mushroom stem waste may be a value-added functional ingredient that can be used at 4% level to improve the nutritional profile, physicochemical properties, and shelf-life of meat products.

## Figures and Tables

**Table 1:** Formulation of goat meat nuggets with different levels of mushroom stem waste (MSW) powder.

Ingredients (%)	Treatment			
	Control	T2	T4	T6
Goat meat	71	69	67	65
Salt	1.5	1.5	1.5	1.5
Water (Ice)	10	10	10	10
Refined oil	8	8	8	8
Condiments *	4	4	4	4
Dry spice mix **	1.8	1.8	1.8	1.8
Wheat flour	3.5	3.5	3.5	3.5
Polyphosphate	0.03	0.03	0.03	0.03
Sodium nitrite (ppm)	150	150	150	150
MSW powder (%)	0.0	2.0	4.0	6.0

Treatments: Control = no additive; T2 = 2.0% MSW powder, T4 = 4.0% MSW powder and T6 = 6.0% MSW powder. * Condiments: fresh garlic and onion (4:1). ** Dry spice mix (18 g/kg nuggets): aniseed, black pepper, capsicum, caraway seed, cardamom, cinnamon, cloves, coriander powder, cumin seed, turmeric and dried ginger (Cookme, Kolkata, India).

**Table 2:** Proximate composition (mean values ± SE) and antioxidant activity of enoki mushroom stem waste powder/extract.

**Proximate Composition (g/100 g Dry Matter)**	
Moisture	12.9 ± 0.3
Protein	13.5 ± 0.7
Fat	1.47 ± 0.04
Ash	8.24 ± 0.05
Total dietary fiber	32.3 ± 0.9
Soluble dietary fiber	17.3 ± 2.1
Insoluble dietary fiber	15.1 ± 2.7
**Antioxidant Capacity of MSW Extract (1 mg/mL)**	
Total phenolics (mg GAE/g)	6.3 ± 2.5
DPPH scavenging (%)	84.2 ± 3.0
FRAP (%)	60.1 ± 1.2
Chelating ability of ferrous ion (%)	41.3 ± 0.5

DPPH: 2, 2-diphenyl-1- picrylhydrazyl; FRAP: ferric reducing antioxidant power; GAE: gallic acid equivalents; *n* = 6.

**Table 3:** Effect of enoki mushroom stem waste (MSW) on the physicochemical, textural and color attributes of goat meat nuggets (*n* = 6).

Parameters	Treatments			
	Control	T2	T4	T6
Emulsion pH	6.33 ± 0.02 ^c^	6.37 ± 0.02 ^bc^	6.39±0.02 ^a^	6.40±0.02 ^a^
Emulsion Stability (%)	94.32 ± 0.20 ^b^	95.86 ± 0.19 ^a^	96.25±0.22 ^a^	96.67±0.22 ^a^
pH	6.38 ± 0.01 ^c^	6.40 ± 0.01 ^bc^	6.42±0.10 ^a^	6.44±0.0.1 ^a^
Cooking loss (%)	5.08 ± 0.12 ^c^	4.12 ± 0.08 ^b^	3.83 ± 0.16 ^ab^	3.12 ± 0.18 ^a^
Total phenolics content (mg GAE/g)	0.142 ± 0.42 ^d^	0.44 ± 0.39 ^c^	0.62 ± 0.45 ^b^	0.96 ± 0.38 ^a^
Expressible water (%)	26.3 ± 1.4	24.2 ± 2.3	22.4 ± 2.4	21.84 ± 2.02
**Proximate Composition (g/100 g)**				
Moisture	65.29 ± 0.54	65.36 ± 0.82	66.74 ± 0.56	67.23 ± 0.56
Protein	15.35 ± 0.24	15.17 ± 0.29	15.22 ± 0.26	14.68 ± 0.20
Fat	12.26 ± 0.29	12.15 ± 0.24	12.13 ± 0.32	12.08 ± 0.12
Ash	2.67 ± 0.05 ^d^	3.54 ± 0.03 ^c^	4.26 ± 0.05 ^b^	4.68 ± 0.03 ^a^
Total dietary fiber	0.82 ± 0.06 ^d^	1.28 ± 0.04 ^c^	1.42 ± 0.06 ^b^	1.72 ± 0.04 ^a^
**Textural Parameters**				
Hardness (N/cm^2^)	42.42 ± 1.86	38.40 ± 1.92	36.33 ± 2.08	34.33 ± 2.12
Springiness (cm)	0.86 ± 0.01	0.84 ± 0.02	0.83 ± 0.02	0.83 ± 0.02
Cohesiveness	0.48 ± 0.02	0.47 ± 0.01	0.45 ± 0.01	0.44 ± 0.01
Gumminess (N/cm^2^)	14.79 ± 1.04	13.42 ± 1.22	12.83 ± 1.34	12.08 ± 1.57
Chewiness (N/cm)	14.05 ± 0.82 ^a^	13.14 ± 0.78 ^ab^	11.75 ± 0.91 ^b^	9.46 ± 0.84 ^b^
**Color Parameters**				
L* value	47.42 ± 0.22 ^c^	48.68 ± 0.19 ^bc^	49.42 ± 0.24 ^ab^	52.02 ± 0.20 ^a^
a* value	7.62 ± 0.20 ^a^	7.48 ± 0.18 ^a^	7.22 ± 0.16 ^ab^	6.32 ± 0.14 ^b^
b* value	13.24 ± 0.32	13.12 ± 0.28	13.18 ± 0.22	13.20 ± 0.24

Treatments: Control = no additive; T2 = 2.0% MSW powder, T4 = 4.0% MSW powder and T6 = 6.0% MSW powder. ^a–c^ Mean values in the same row bearing different superscript differ significantly (*p* < 0.05).

**Table 4:** Effects of enoki mushroom stem waste (MSW) powder on the sensory characteristics of goat meat nuggets (*n* = 30).

Parameters	Treatments			
	Control	T2	T4	T6
Appearance	7.03 ± 0.12	7.07 ± 0.10	7.00 ± 0.14	6.94 ± 0.12
Texture	6.88 ± 0.13	6.92 ± 0.16	7.01 ± 0.10	6.96 ± 0.14
Flavor	6.84 ± 0.10	6.90 ± 0.18	6.86 ± 0.16	6.80 ± 0.14
Juiciness	6.74 ± 0.11	6.89 ± 0.13	6.98 ± 0.12	6.95 ± 0.12
Overall acceptability	6.87 ± 0.14	6.90 ± 0.16	6.98 ± 0.18	6.84 ± 0.20

Treatments: Control = no additive; T2 = 2.0% MSW powder, T4 = 4.0% MSW powder and T6 = 6.0% MSW powder.
